# Happiness

**Published:** 1870-07

**Authors:** 


### HAPPINESS.

To be truly happy a man must be healthy and good; a sick man may be happy in some transient moods of mind, but this is of short duration. To have a sustained happiness, to have in the main, from day to day, a life of sunshine, good health is indispensable. But if a man is sick in body, and unhappy in his mind, how then? Procure a constant occupation in which you take a pleasurable interest while you are reasonably sure that you are accomplishing your object. If this is an out-door occupation, success is the more speedy and certain. It ought always to be borne in mind that whatever is done for the health, will be many fold more efficient if an object is kept in view, so absorbing, that the health part is kept completely out of sight.

